# Analysis of Methods for Long Vehicles Speed Estimation Using Anisotropic Magneto-Resistive (AMR) Sensors and Reference Piezoelectric Sensor

**DOI:** 10.3390/s20123541

**Published:** 2020-06-22

**Authors:** Vytautas Markevicius, Dangirutis Navikas, Donatas Miklusis, Darius Andriukaitis, Algimantas Valinevicius, Mindaugas Zilys, Mindaugas Cepenas

**Affiliations:** Department of Electronics Engineering, Kaunas University of Technology, Studentu St. 50–439, LT-51368 Kaunas, Lithuania; vytautas.markevicius@ktu.lt (V.M.); dangirutis.navikas@ktu.lt (D.N.); donatas.miklusis@ktu.edu (D.M.); algimantas.valinevicius@ktu.lt (A.V.); mindaugas.zilys@ktu.lt (M.Z.); mindaugas.cepenas@ktu.lt (M.C.)

**Keywords:** magnetic field measurement, sensors, cross-correlation, vehicle speed estimation, AMR, long vehicles

## Abstract

With rapidly increasing traffic occupancy, intelligent transportation systems (ITSs) are a vital feature for urban areas. This paper analyses methods for estimating long (L > 10 m) vehicle speed and length using a self-developed system, equipped with two anisotropic magneto-resistive (AMR) sensors, and introduces a method for verifying the results. A well-known cross-correlation method of magnetic signatures is not appropriate for calculating the vehicle speed of long vehicles owing to limited resources and a long calculation time. Therefore, the adaptive signature cropping algorithm was developed and used with a difference quotient of a magnetic signature. An additional piezoelectric polyvinylidene fluoride (PVDF) sensor and video camera provide ground truth to evaluate the performances. The prototype system was installed on the urban road and tested under various traffic and weather conditions. The accuracy of results was evaluated by calculating the mean absolute percentage error (MAPE) for different methods and vehicle speed groups. The experimental result with a self-obtained data set of 600 unique entities shows that the average speed MAPE error of our proposed method is lower than 3% for vehicle speed in a range between 40 and 100 km/h.

## 1. Introduction 

Recent technological advances have revolutionized intelligent transport systems (ITSs) in terms of data collection, traffic management, and control. Information obtained by ITSs is necessary to provide various services that enable smoother, safer, and environmentally friendly transportation. Scientists have investigated various vehicle detection methods, ranging from inductive loops, piezo/quartz, and air switches/road tubes to various magnetometers, microwave radars, passive infrareds, ultrasonic, and video surveillance systems [1,2,3].

The integrated magnetic field sensor is superior to other sensing technologies owing to its unique advantages, such as being insensitive to climate and weather conditions, being less susceptible than loops to stresses of traffic, its low cost, its small volume, and its comparability to wireless communication [4]. The use of this technology is mostly based on the anisotropic magneto-resistive (AMR)-type sensor. An obtained magnetic signature captures vehicle features and could be used for estimating speed and length and performing vehicle classification [5]. Cross-correlation is a base method for accurate speed estimation from a pair of magnetic signatures.

However, in the case of long buses and trucks (length > 10 m), passing at relatively low speeds (30–60 km/h), the length of the magnetic signatures can accumulate up to 10,000 and more samples. In this case, the calculation of cross-correlation requires large computational resources, energy, and time [6,7]. As a result, the system becomes expensive and energy-inefficient, and miscalculates the volume and speed of traffic. Wrongly estimated vehicle speeds cause incorrectly calculated lengths and erroneous classification.

In this paper, we proposed a reference method to verify the accuracy of speed estimation. An additional video camera and the piezoelectric polyvinylidene fluoride (PVDF) sensor mounted into a pavement of road together with the AMR sensors provide a ground truth speed.

This study is follow-up research for the previously described experimental setup [8]. This publication aims to expand the proposed system monitoring capabilities for all road traffic members. The dataset of 600 unique entities was collected from real traffic conditions and different speed estimation methods were compared, focusing on magnetic signatures of long vehicles. In order to reduce power consumption and to simplify data processing, novel signal cropping algorithms were proposed and subsequently evaluated.

## 2. Related Works 

Conventional traffic monitoring systems are carried out with the use of detectors that need to be installed in the pavement (e.g., inductance loops, piezoelectric sensors) or need a supporting structure (video surveillance systems and microwave radars). The vision-based traffic monitoring system has the advantages of high detection accuracy, monitors multiple lanes and multiple detection zones, provides a rich array of data, and is widely used in the word. However, a video vehicle detector requires periodic lens cleaning, performances are affected by inclement weather, it cannot detect axles, and it requires high computation power [9]. Inductive loops cause severe intrusion into a pavement, require lane closure, and commonly do not detect axles. Although the aforementioned technologies are mature and well-known, the rapidly growing transportation sector raises new requirements. Furthermore, owing to high installation and maintenance prices, low-cost sensing technologies that enable easy installation and maintenance are receiving more attention [2].

### 2.1. Alternative Vehicle Sensing Detectors

A sensor node for traffic monitoring made of a combination of polytetrafluoroethylene (PTFE) and aluminium film deployed across the road was presented by Yadav et al. [3]. In that solution, a well-known triboelectric effect was utilized to generate voltage spikes owing to frictional contact among two materials. An important advantage of this kind of sensor is that using them in pairs allows to measure vehicle speed owing to the time difference between the reception of voltage signal, while the magnitude of a signal can be utilized for weight measurement. For the proposed method, the mean absolute percentage speed error (MAPE) is 3% and it was found that, for weight estimation, the obtained voltage signal varies linearly with vehicle speed.

The Ma et al. [10] have investigated an automatic vehicle classification system targeting heavy vehicles with multiple axles. The network of accelerometers was used to detect axles of a vehicle, while the readings of magnetometers were used for estimating speed. In the introduced traffic monitoring system, data from wireless sensors were collected by the nearby access point and analyzed. With 1.2 m spacing between sensors and a cross-correlation calculation of magnetic signatures, the authors reached a speed error of ±1.6 km/h. Furthermore, the proposed solution allows vehicle classification according to axle number with accuracy close to 99%.

Another similar approach conducted by Ye et al. [11] with only vibration sensors shows ±4% relative speed error and the authors declare 100% classification precision for a group of long vehicles.

In the related research of Lou et al. [12], efforts have been made to tackle a multiple vehicle detection problem, caused by a magnetic sensor blind zone. This zone appears between the front and rear axle of high-chassis vehicles such as trucks, which leads to one long signature classification as two passenger cars. The authors had introduced the idea to use received signal strength from radio stations to add additional information about the traffic state. The experimental results show that the proposed method can reach up to 12% better detection accuracy for long vehicles compared with single magnetism-based detection. A similar approach that utilizes only radio signals is presented in the work of Sliwa et al. [13]. The multidimensional attenuation patterns of wireless signals are used for vehicle classification, speed, and length estimation. The authors presented a prototype system of six communicating sensor nodes and declared classification accuracy of 93% with seven classification groups.

An alternative technique for detecting vehicles is sensors employing optical fiber described by Liu et al. [14]. There are different working principals of this kind of sensor, but generally, the measurement is based on changes in the fiber refraction index owing to mechanical stresses. As light is exploited in this approach, it has many significant advantages, such as resistance to corrosion, being immune to electromagnetic interference, high sensitivity, remote operation, and multiplexing capability. On the other hand, these kinds of sensors need to have almost a physical contact with the sensed body; a single node cannot estimate the vehicle speed and it requires difficult and expensive equipment to inject, receive, and analyze the signal.

### 2.2. Magnetic Sensors

Studies have shown [1,2,12] that the magnetic sensor for the intelligent transportation system is superior owing to its unique features, such as its small volume, being immune to climate and weather conditions, suitability for wireless communication, and relatively large sensitivity. With every new generation, a magnetic field sensor becomes more robust, compact, precise, and affordable. Although, other types of magnetic field sensors (e.g., tunnel magneto-resistance (TMR) and giant magneto-resistive (GMR)) are gaining popularity in vehicle detection systems, where the most commonly applied are AMR type sensors [15,16,17]. One advantage of using AMR sensors is the relatively wide output signal dynamic range. It allows to simplify the circuitry and connect sensor output directly to a microcontroller analog-code converter.

As most of the research shows, there are mainly two reliable methods for speed estimation using magnetic vehicle signature: (1) normalized cross-correlation calculation of a pair of obtained signals or (2) various segmentation of signals into ROI (regions of interest) and delay estimation according to a threshold value.

The cross-correlation method provides the best overall accuracy [16] and further ensures the low possibility of outlier’s readings. As long as a signature shape is not distorted, this method provides a correct delay between signals. Many ways of calculating cross-correlation for vehicle speed estimation have been proposed. Some of them use a single component of a magnetic field [4], a magnitude of all components [17], and a derivative signal of magnitude [18], among others. On the other hand, as it is based on multiple multiplication and aggregation, it needs relatively large computing power.

The alternative option is to use threshold-based methods. The challenge is how to ensure identical sensitivity and synchronization among two or more sensors. Most of the methods consist of division of complex original signals into sub regions and adaptive threshold calculation [19]. The third option is to combine assets of the above-mentioned methods in order to obtain the same accurate result as with cross-correlation and ensure fast computation as with the threshold method.

The Zhu et al. [7] have proposed to use state machine detection (SMD) in conjunction with cross-correlation detection. This method employs two lateral displaced sensors to start obtaining signals only when SMD triggers a vehicle. It is done using adaptive threshold value calculation with state machine design for canceling the interference of noises. Besides, the authors propose cross-correlation calculation of original and reference signal, obtained after segmentation and k-mean clustering. As a result, it provides a better resolution of detecting the moment of arriving or departing vehicles. Although the authors provide promising results, the reference signal was obtained by applying a training algorithm with a certain amount of data and does not fit all cases.

A unique approach has been analyzed using the dynamic time warping (DTW) method by Burresi et al. [20]. It is based on finding the best match of the first signal point into the second signal previous and successive instants. However, the authors pointed out that this method does not provide significant improvements in the alignment of two curves.

A comparative analysis of a stationary traffic monitoring station was carried out in previously published articles [6,8]. The authors focused on analyzing sources of speed and length estimation errors regarding a distance between two sensor nodes placed on a road lane. In the work of Markevicius et al. [8], speed estimation method based on cross-correlation of the first order derivative of the magnetic signature was presented and tested with a dataset of 250 vehicles. An important part of the paper is the analysis of speed estimation error owing to the increment Δn used in numeric differentiation of magnetic field magnitude data. Although the paper provides promising results, there is a need to compare the proposed speed estimation method with other methods, use a larger dataset, and include vehicles from all groups. Furthermore, computation complexity and energy consumption should be taken into account.

## 3. Problem Definition

With the ongoing development of intelligent transportation systems, requirements for a single node speed measurement device are expanding to interconnected traffic monitoring and controlling network. It is desired to obtain real time information about traffic flow, classify vehicles by type, and distribute data acquisition nodes through urban areas. A magnetic field sensor together with a sophisticated digital signal processing stage provide unique data of traffic that is on study demand [4,6].

Regarding system accuracy for speed estimation, it is preferred to have as a high sampling frequency (fs) as possible. On the other hand, it complicates the data processing part. Especially, in situations with long buses and trucks, the recorded signal at 2 kHz fs could have up to 10,000 samples and more. Since the data processing part needs to be handled by a low-power consumption controller, the additional data segmentation stage needs to be implemented.

Although studies [17,20] show that the cross-correlation method of a full magnetic signature provides the most accurate speed estimation results, owing to the aforementioned reason, it is not always suitable. Nevertheless, it should be underlined that it is not possible to obtain a reliable time difference value from signals with only an ascending (descending) part. In other words, the signal segmentation algorithm must be applied very carefully to adaptively crop out at least one cycle of ascending and descending.

The idea of using a derivative signal of magnetic field sensor readings, instead of a magnitude signal, to calculate the speed is quite attractive owing to the number of features. Unlike the magnitude signal, the derivative signal crosses the time axis multiple times and is convenient for splitting signature for further cross-correlation calculation.

## 4. Vehicle Speed and Length Measuring System

**Figure 1:**
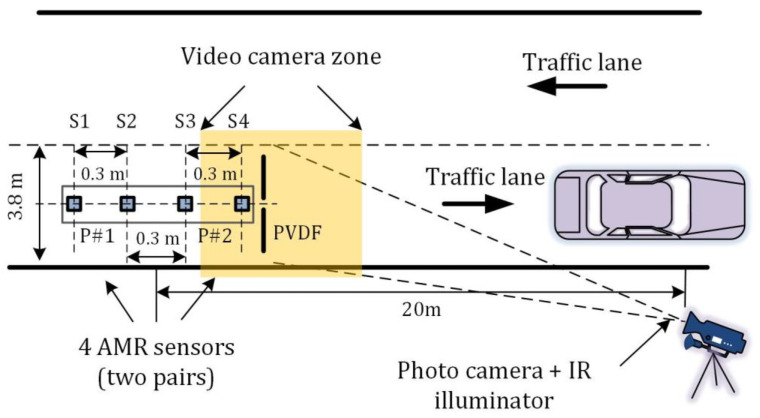
Vehicles speed measurements system prototype. It is equipped with four anisotropic magneto-resistive (AMR) magnetic sensors, a traffic surveillance camera, and polyvinylidene fluoride (PVDF) sensors for estimating ground-truth speed [8].

The measuring system diagram is depicted in Figure 1. Our developed system consists of four AMR (LIS3MDL, STMicroelectronics Inc., Santa Clara, CA, USA) sensors installed in the pavement of a road. Each of the sensor nodes is equally spaced by 0.3 m. This allows us to perform a calculation with different gap distances between two nodes. While the larger gap provides better time resolution, near placed sensors records more identical curvature signals. After a magnetic sensor detects a vehicle inside of the shooting zone, the traffic surveillance camera is triggered.

**Figure 2:**
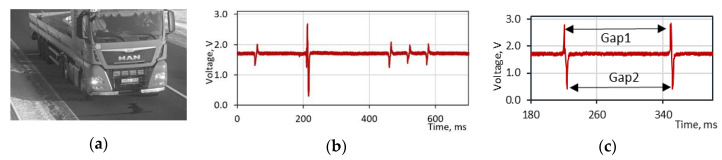
(**a**) Five-axle truck. (**b**) PVDF sensor raw discretized signal of the five-axle truck. (**c**) The time difference between two adjacent peaks is calculated by the average of the upper and lower gaps.

For a result validation, an additional reference system is installed together with the magnetic sensors. The polyvinylidene fluoride (PVDF) film sensors are placed perpendicular to the traffic direction. An analog output signal is digitized by a 2 kHz sampling frequency ADC converter. A time domain signal contains data of vehicle axles number, while the amplitude of signals could be used for weight in motion applications. For a speed estimation, we are interested only in the time difference between two neighboring peaks (Figure 2).

**Figure 3:**
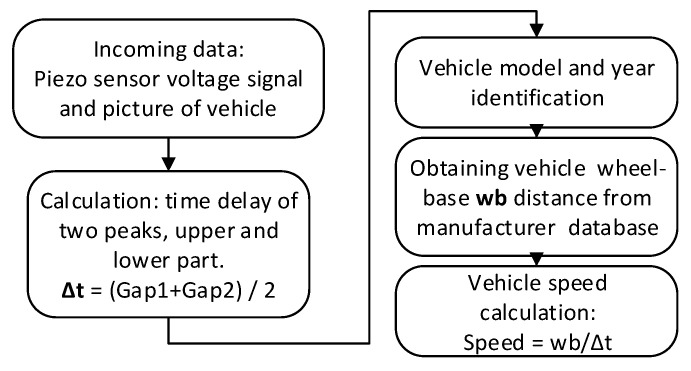
Functional diagram of the reference system.

The functional structure of the reference system is depicted in Figure 3. First, the time gap between two sequentially peaks is obtained, using PVDF sensor readings. Then, a vehicle is identified from a picture and a wheelbase (wb) distance is acquired from a manufacturer database.

The estimated speed error from the reference system depends on two parameters: estimated time difference between two peaks and wheelbase. As the PVDF readout circuit works at 2 kHz sampling frequency, peaks’ location is identified with ±0.5 ms uncertainty. The second parameter’s error depends on a manufacturer database, which varies with different brands. Considering margins, we assumed that a wheelbase length is provided with the uncertainty of ±10 mm. With those initial conditions, one could estimate that a vehicle traveling at 50 km/h speed could be recorded with ±0.3 km/h absolute error (±0.7% relative error), and vehicle at 100 km/h with ±1.0 km/h (±1.0% relative error) (Table 1).

Using the target and the reference systems simultaneously, the signatures’ dataset for signal processing were accumulated. Each entity of the dataset consists of a unique number, the speed value from the reference system, and the raw magnetic signatures from four sensors. According to a vehicle length, the dataset is divided into four groups (Table 2). After a particular period of recording, over 600 unique entities were obtained.

## 5. Speed Estimation

On the basis of experience from the previous experiments [6], it was chosen to use magnetic signal magnitude versus the only absolute value of the z-component for vehicle speed estimation. In this paper, five speed estimation methods are proposed and verified. Each method is explained in detail below, and they were all tested with the acquired dataset and compared in the following result section. Method 1 and 2 are threshold-based approaches to detect arrival and departure points. A vehicle is stated as detected if a sensor reading is larger than the chosen threshold value. A magnetic signature magnitude signal and its first order derivative are used with these methods. The following three methods (3, 4, 5) are based on calculation of the cross-correlation function. The unique feature of method 5 is the signal cropping algorithm, employed in order to reduce the complexity of cross-correlation calculation. 

Furthermore, signal conditioning plays a significant part in the estimated results. It was noted that, depending on a vehicle type and position regarding the magnetometer sensor, a recorded signal has a wide dynamic amplitude range. Although most vehicles produce signals with significant signal-to-noise (S/N) contrast, there are some signatures with a relatively low S/N level. These kinds of signals with speed estimation methods provide false results as a threshold level is falsely triggered by a noise level. Therefore, the filtering influence with every method has been investigated. Lowering the cut-off frequency (fc) of a low-pass filter (LPF) leads to better de-noising performance, although it results in loss of temporal resolution, which degrades the ability to precisely localize signature feature points. These opposed constraints mean that the best fit fc frequency differs for every method.

### 5.1. Threshold-Based Method 1 and Method 2

**Figure 4:**
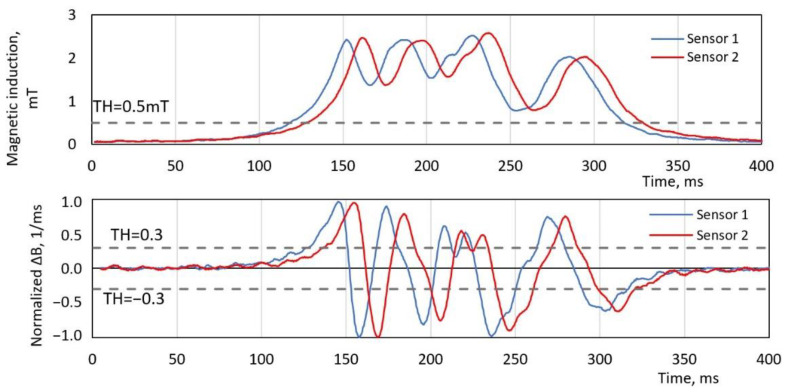
An example of two sensor readings with speed estimation method 1 and method 2. The threshold value (TH) for the magnetic magnitude signal (**top**) and for the first derivative of the magnetic magnitude (**bottom**).

Using these two methods, speed estimation is based on the time delay between two signals at the threshold value (Figure 4) position. As both of the sensor nodes are placed along a vehicle moving direction, the pair of signals is shifted just in time. Therefore, it is feasible to use one common threshold value to find the time delay between the signals. For the second method, the normalized first order derivative of the signal was used. Using normalized values, it eliminates the necessity to use signals with identical amplitude levels. For both methods, the best-fit threshold value in the pair with an LPF cutoff frequency was obtained by the trial method, minimizing an average speed error. The obtained results are provided and explained in the results section.

### 5.2. Method 3 and Method 4

The following two methods (3, 4) are based on the location of the maximum value of the cross-correlation function in the time domain. The difference among them is the pair of input signal vectors. For method 3, magnetic signature magnitude is used and, for method 4, the first order derivative of the magnetic signature is used.

### 5.3. Method 5. Cross-Correlation of the Fraction of the First Order Derivative of the Magnetic Signature Magnitude

Although the cross-correlation method provides the best overall accuracy, it requires relatively high computing power and speed cannot be estimated before obtaining a full signature from both nodes. For passenger vehicles, it is not an issue, but for long trucks and buses (L > 10 m), there are many benefits to knowing speed before they have passed over the sensor. First, a standard euro truck of 16 m length and with 70 km/h speed needs an 822 ms period to overdrive the sensor. For this amount of time, sensors must be on, acquire samples, and store measurements in sufficient size temporal buffers. Secondly, for speed measurement systems with a camera, it is necessary to estimate vehicle location in the near future for the best picture in focus. In order to meet those requirements for long vehicles, sensors must be placed at a sufficient distance in advance before the camera, although it increases the uncertainty of shooting time. Therefore, it was proposed to use only a fraction of a magnetic signature.

**Figure 5:**
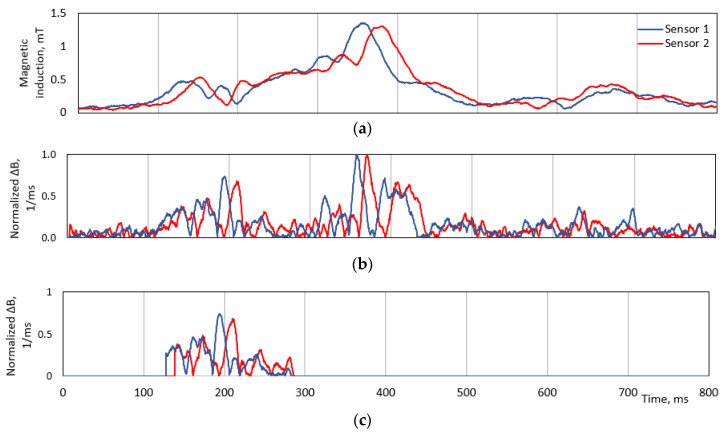
An example of signals cropping algorithm with the windows method. (**a**) An original magnetic field magnitude. (**b**) The normalized module of derivative of magnitude. (**c**) The result of the signature cropping algorithm—a pair of fractions of derivative signals.

On the other hand, it is not straightforward to crop part of a signal for calculations. Cross-correlation requires to use two identical curvatures, shifted in time signals. In order to crop two identical parts, it is necessary to recognize the feature points. As a derivative signal crosses zero level multiple times, it is more practical to use those points as references (Figure 5).

Two cropping algorithms are proposed in this paper:

1. Cropping according to a minimum width of window;
2. Cropping according to a peak number of the first order derivative signal.

**Figure 6:**
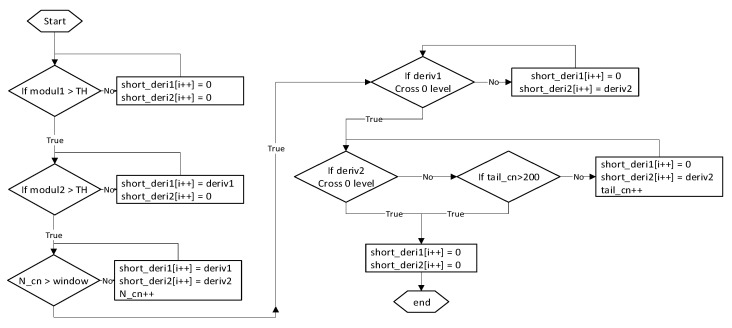
A signature cropping algorithm by window method, where modul1(2)—vector of magnetic signature magnitude, deriv1(2)—vector of difference quotient of magnetic signature, short_deriv1(2)—vector of cropped derivative signal, TH—threshold value, N_cn—counter of signal vector element, window—window method parameter, tail_cn—counter for forcing the end of the recording, and i++—index of vector element.

The first algorithm is depicted in Figure 6. There are a few parameters used for this method: window width (minimal amount of data points to crop), TH (threshold for magnetic magnitude), and tail counter (maximum amount of points after window width). The cropping is triggered if the magnetic signature magnitude exceeds the TH value. After both signals have crossed the TH value, a short buffer keeps being filled until the amount of points exceeds the window width parameter. In the last stage, the algorithm tracks when each of the derivatives of signal crosses the zero level and stops each channel separately. If for some reason, the signal does not reach zero level, there is a tail counter, which forces the sampling to stop.

The main drawback of the first method is not an adaptive window width. Two vehicles with different speeds would provide unequal curvature patterns and, for further calculations, a signal with multiple peaks is better than just a slow ramp-up signal. Therefore, as an adaptive alternative to the first algorithm, it was proposed to count peaks of a derivative signal and crop a particular number of those. This method is speed invariant and it would crop the same curvature pattern signal, despite the speed of a vehicle. The overview of the results is provided in the following section.

## 6. Result and Accuracy

All five proposed methods were verified with a dataset from a self-developed monitoring system, which is described in Section 4. Estimated speed values were compared with reference values and a mean absolute percentage error (MAPE) criterion was used; Equation (1). As it is expected to have an outlier in the result dataset owing to errors in the measuring system, the root mean square error (RMSE) criterion is undesirable owing to its high weight on large errors.

(1) $$\mathrm{MAPE}=\frac{100\%}{M}\overset{M}{\underset{m\;=1}{\sum }}\left|\frac{V_{\mathrm{AMR}}-V_{r}}{V_{r}}\right|,$$

where M—number of vehicles in the dataset (600 samples), V_AMR—estimated speed from AMR sensors, Vr—reference speed from PVDF sensors, and m—vehicle number in the dataset.

### 6.1. Threshold-Based Speed Estimation

**Figure 7:**
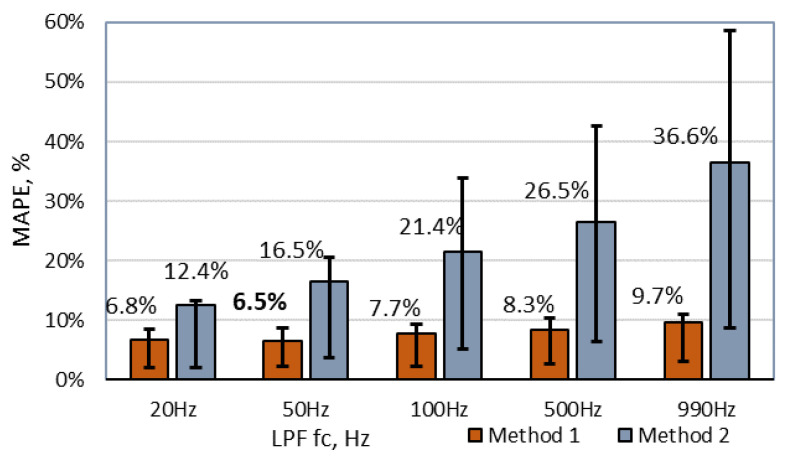
An estimated speed mean absolute percentage error (MAPE) for methods 1 and methods 2 versus applied low-pass filter (LPF) with the particular cut-off frequency with a constant threshold level. Error bars indicate a percentage error range of second and third quartiles. fc, cut-off frequency.

The first two methods that run multiple times with a different combination of a threshold value in a pair of applied filter cut-off frequency and the results are compared in Figure 7. The MAPE of all dataset samples is plotted versus LPF cut-off frequency with a constant threshold value. It is visible that, for the best performances, filtering with at least fc = 50 Hz needs to be applied. Further, it is obvious that the threshold method with a magnitude-based signal performs much better than a derivative signal. The lowest MAPE value of 6.4% was obtained with a magnitude signal at 50 Hz filtering and a threshold value of TH = 0.5 mT, which refers to five times the ground noise level. Meanwhile, the lowest error of method 2 with a derivative signal was only MAPE = 13.6% at Th = 0.3 for normalized signals.

Furthermore, an error bar in the figure indicates a range of second and third quartiles of all samples in the database. As shown, method 2 has a significant part of outlier measurements, the number of which was highly reduced by reducing LPF fc frequency. In the case of method 1, a filtering part helped to reduce the average error level from 9.7% to 6.5%.

### 6.2. Results of Cross-Correlation Methods

**Figure 8:**
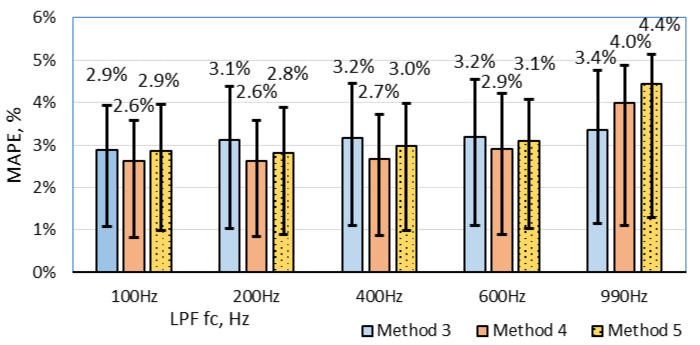
An estimated speed MAPE as a function of LPF cut-off frequency with three different methods. Error bars indicate a percentage error range of second and third quartiles.

The cross-correlation results are shown in Figure 8. Three different methods are analyzed: magnitude-based, derivative, and fraction of the derivative. The filtering part has a significant impact on the results. The best results are obtained with 100–200 Hz LPF. A filter with a lower cut-off frequency than 100 Hz filters out important features of signature and errors increase. Cross-correlation with a derivative signal performs better than a calculation based on magnetic signal magnitude. Using filtering, MAPE was reduced from 4.0% to 2.6%. Furthermore, the method with the fraction of the derivative signal shows a similar result like a full derivative, but the calculation time for this method is significantly lower. It could be said that all cross-correlation methods provide a twofold better result than threshold-based methods.

**Figure 9:**
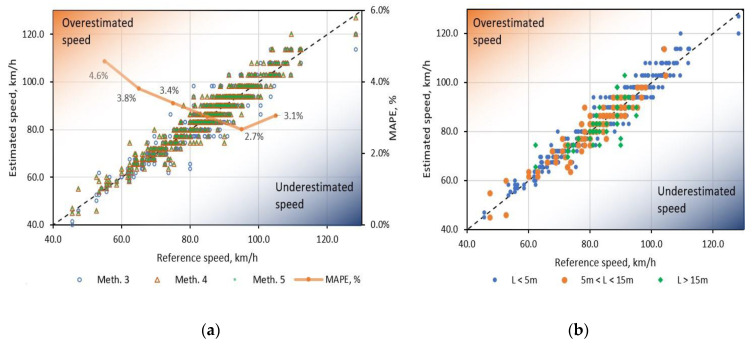
(**a**) Speed estimation performances of methods (1, 2, 3) and MAPE versus reference speed from the PVDF sensors. (**b**) Speed estimation performances with method 3 versus three different vehicle length groups (L < 5 m, 5 m < L < 15 m, and L > 15 m).

In Figure 9a, the estimated results of methods (1, 2, 3) are plotted versus speed values from the reference system. It is visible that all methods are distributed closely together and have comparable results. Furthermore, an average MAPE of method 5 was calculated for speed bins with a width of 10 km/h and plotted in the background of the chart. It is visible that minimum MAPE = 2.7% was achieved within the speed range [90–100 km/h]. Similarly, in Figure 9b, the speed estimation results are plotted with three different vehicle length groups: L < 5 m, 5 m < L < 15 m, and L > 15 m. One could see that all the results are similarly propagated and there is no significant offset difference between long and average length vehicles.

### 6.3. Accuracy Enhancement of an Estimated Speed

As shown in the work of Lai et al. [21], the accuracy of an estimated speed value by the cross-correlation method could be increased by applying interpolation. The main drawback of this approach is significantly increased calculation time. In this paper, we propose a different method for increasing estimation accuracy without introducing complexity. Firstly, the cross-correlation maximum is found. Then, using four data points around this maximum, the best fit cubic function is obtained. The maximum of this function refers to a fractional delay between signals. As shown in Table 3, with all three methods, the accuracy enhancement technique helped to reduce MAPE error by [0.1–0.2%]. Owing to a finite signal sampling frequency, estimated speed uncertainty error increases with speed. Therefore, the proposed enhancement technique is more significant with a higher speed vehicle group.

### 6.4. An Estimated Speed Error Owing to Recording Window Width

The previous section shows that method 5 with a fraction of signature provides similar results as a method with a full signal. A shorter sampling period has a significant impact on system energy performances. It allows to operate hardware in low power mode, while most of the time, the device is in an idle mode and sampling happens only for a short period of time. Further, it requires a smaller buffer size and reduces the need for computing power. This is a big advantage in the case of long and slow-moving vehicles.

**Figure 10:**
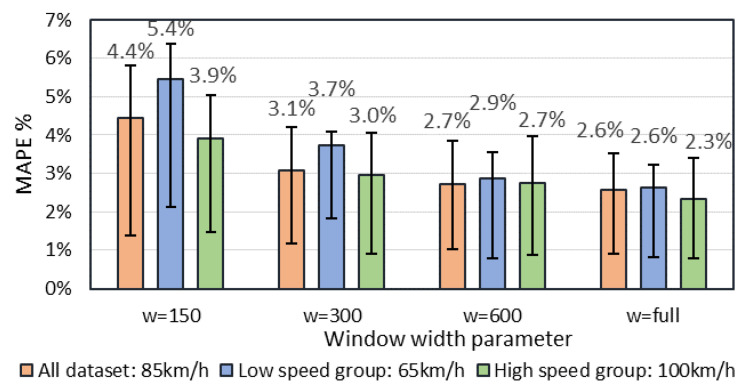
Speed error MAPE versus cropping algorithm window parameter for three different average speed groups. Error bars indicate a percentage error range of second and third quartiles.

In order to find a sufficient minimum sampling period, method 5 was investigated with a variable window size parameter, which describes a minimum number of signature data points. As the sampling period is 2 kHz, a 300-point vector represents a 150 ms period signal. In order to characterize a result with respect to a reference speed, three subgroups were analyzed: vehicles at high speed (average speed 100 km/h), low speed (average speed 65 km/h), and a mix of all dataset records (average speed 85 km/h). The length of magnetic signature depends on speed and, for fast moving vehicles, the average length is 560, while for slow moving vehicles, it is 960 points. Four different windows parameters were analyzed (Figure 10), starting with the full signature to signal with 150 data points. As shown, a speed estimation error increases by reducing the signature length. At the shortest window size, slow moving vehicles have the highest error value. This is because of low feature points in an analyzed fraction of the signal. On the other hand, a window of 300 data points shows the best performances. On average, it provides about a 50% shorter signal, which decreases cross-correlation calculation complexity by a factor of 4. Additionally, with the window parameter of 300, an estimated speed MAPE for all dataset vehicles is just 0.5% higher than using a full signal.

### 6.5. Derivative Signal Cropping and Calculation Complexity

**Figure 11:**
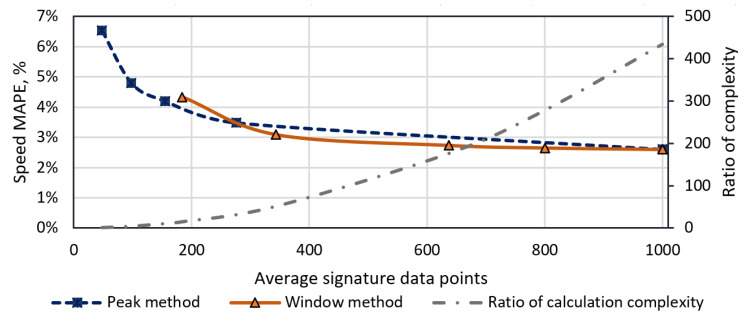
Comparison of signature cropping methods. Estimated speed MAPE of window and peak counting methods versus cropped data points of signature and relative computation complexity versus signature length.

The second cropping algorithm was also tested with the whole dataset. It is visible in Table 4 how many points on average the cropped signal has versus a different parameter value. The lowest parameter value is one peak, which represents a signal with only one peak. The computation complexity of the two cropping algorithms is depicted in Figure 11. The chart shows that, by increasing the cropped algorithm from one to five peaks, the average amount of signature data points increases from 48 to 276, and the estimated speed error MAPE decreases by 3%. Further signature length increment benefits just by 1.1%. On the other hand, cross-correlation calculation complexity increases by a power of 2 with respect to signal length. Therefore, calculation complexity using a signature with five peaks versus full signal differs by a factor of 13.

In order to compare the proposed cropping algorithm benefit regarding execution time, speed estimation methods were run on corresponding STM32F767ZI MCU working at the frequency of 120 MHz. Cross-correlation calculation was executed using two discrete-time sequences of 343 and 1000 samples each, which represent a cropped signal with a window parameter w = 300 and a full signature of a long vehicle. The measurements show that a shorter signal was processed in 26.5 ms and a longer signal in 224.4 ms. It is worth mentioning that the cropping algorithm not only reduces the calculation time by the factor of 8.5, but also allows to start calculation before a vehicle passes over the sensing zone. For instance, if the starting time is the vehicle arrival moment, methods 3 or 4 provide an estimated speed value only after ~ 730 ms (500 ms + 224.4 ms), in the case with a signature of 1000 samples. Whereas, because method 5 runs in parallel with the signal logging part, the result could be provided after only ~ 200 ms (172 ms + 26.5 ms).

## 7. Discussion

First of all, it is worth noting that a signal filtering with LPF has a significant impact on the estimated speed error and best-fit cut-off frequency varies from a method. Speed estimation methods based on a single threshold value crossing (method 1 and method 2) require the least computation power and are easiest to implement. Although, they provide the poorest result as well. The minimum mean absolute percentage error (MAPE) for method 1 is 6.5% and, for method 2, it is 13.6%. Additionally, both methods work best with low fc frequency (<50 Hz).

The second analyzed speed estimation technique is cross-correlation. For the methods (3, 4, 5), the minimum MAPE values are 3.1%, 2.6%, and 2.8%, respectively. All three of these methods have more than twofold smaller error compared with the best result from the threshold methods (the error is higher by 3.9%). In contrast to the threshold methods, the LPF filtering stage helped to reduce error only by1.4%. On the other hand, applying the LPF stage with cut-off frequency below 200 Hz produces signals with fewer feature points, which consequently increase the error level.

The estimated speed accuracy enhancement method, based on cubic interpolation, shows a positive result. As shown in Table 3, the estimated speed error (MAPE) was reduced by 0.2%. Furthermore, it is visible that this method is more significant in a case with a higher speed vehicle group. This is because of the finite sensor sampling frequency. Therefore, this method allows reducing the error range of sampling uncertainty without physically increasing sampling frequency.

Finally, speed estimation methods with a fraction of a signal show promising results. Using method 5 with a window parameter of 300, which represents the minimum amount of data points in a signal, the minimum MAPE value is only slightly higher, compared with MAPE of full signal cross-correlation, by 2.8%, and 2.6%, respectively. On the other hand, from Figure 11, it is visible that, on average, this method reduces sampling period by a factor of 3 and leads to lower cross-correlation computation complexity by a factor of 10. Furthermore, the signal cropping method with a peak counting algorithm is adaptive to the speed of a vehicle. It provides a fraction of difference quotient of magnetic signature with the same amount of feature points invariant to speed. In Figure 11, it is shown that the first-order derivative signal with five peaks is sufficient for the reliable speed estimation.

## 8. Conclusions

In this paper, a novel speed estimation algorithm for long vehicles is proposed based on a pair of magnetic sensors and evaluated with reference piezo-electric sensors. The proposed approach is enabled by two nodes of the magnetic field sensor spaced by 0.3 m, which are deployed on an intercity two-lane road, where average traffic speed is 85 km/h. Various speed estimation methods are investigated and tested with a dataset from a self-developed monitoring system, which contains 600 unique entities. For the proposed speed estimation algorithm, we presented a digital signal processing part, an adaptive magnetic signature cropping method, which is based on the first-order derivative and cross-correlation with a reduced signal. Our presented framework aims to operate with a low-power consumption microcontroller and wireless communication interface.

The best method results show that an average estimated speed MAPE = 2.6% was achieved, considering vehicles in a speed range from 45 km/h up to 120 km/h. For comparison, a similar study with magnetic field sensors spaced by × 20 larger distance (L = 6 m) achieved RMSE = 5.1 km/h and MAPE = 2.65% speed error [16,22]. The authors of [19] declare speed MAPE = 1.2% with sensors spaced by L = 6 m and a novel region-based processing method.

Furthermore, the adaptive signature cropping algorithm was proposed, which could be employed to estimated vehicle speed before it totally drives over the sensor. It allows reducing computation complexity and monitoring the period of time without degrading the accuracy of the result.

## Figures and Tables

**Table 1:** Uncertainty estimation of the reference system.

Real Speed, km/h	Wheelbase ± Uncertainty, m	Time Gap ± Uncertainty, ms	Estimated Speed ± Uncertainty, km/h	Relative Error, %
50 km/h	3.7 ± 0.01 m	266 ± 1 ms	50 ± 0.3 km/h	±0.7%
100 km/h	3.7 ± 0.01 m	133 ± 1 ms	100 ± 1.0 km/h	±1.0%

**Table 2:** Number of signatures by groups in the dataset.

Vehicle Type	Length Group	Number of Signatures
Passenger cars	L < 6 m	452
Minibuses	6 m < L < 10 m	88
Trucks and buses	10 m < L < 17 m	46
Long trucks (truck with trailer)	L > 17 m	18

**Table 3:** Accuracy enhancement technique performances with three different speed estimation methods and different speed groups. MAPE, mean absolute percentage error.

Speed Range Group										
		All Datasets (Average Group Speed 85 km/h)			Lower Speed Vehicles (Average Group Speed 65 km/h)			Higher Speed Vehicles (Average Group Speed 100 km/h)		
		Meth-3	Meth-4	Meth-5	Meth-3	Meth-4	Meth-5	Meth-3	Meth-4	Meth-5
MAPE %	Original	3.1%	2.6%	2.8%	2.7%	2.5%	2.9%	3.0%	2.4%	2.6%
	With enhance-ment	3.0%	2.5%	2.7%	2.6%	2.4%	2.9%	2.9%	2.2%	2.4%
	Reduction of error	0.1%	0.2%	0.1%	0.1%	0.1%	0.0%	0.1%	0.2%	0.2%

**Table 4:** An estimated speed MAPE comparison of the two cropping methods.

**Window Method**	Parameter	w = 150	w = 300	w = 600	w = 700	full
	Average signature data points	184	343	637	800	1000
	MAPE	4.3%	3.1%	2.7%	2.6%	2.6%
**Peak Counting Method**	Parameter	1 peak	2 peaks	3 peaks	5 peaks	full
	Average signature data points	48	98	155	276	1000
	MAPE	6.5%	4.8%	4.2%	3.5%	2.6%
